# N-Lobe of TXNIP Is Critical in the Allosteric Regulation of NLRP3 *via* TXNIP Binding

**DOI:** 10.3389/fnagi.2022.893919

**Published:** 2022-06-02

**Authors:** Fengyu Cheng, Nan Wang

**Affiliations:** Jiangsu Key Laboratory of Brain Disease Bioinformation, Research Center for Biochemistry and Molecular Biology, Xuzhou Medical University, Xuzhou, China

**Keywords:** NLRP3, thioredoxin interacting protein (TXNIP), N-lobe, allosteric activation, molecular dynamics simulation

## Abstract

Inflammasomes are cytoplasmic complexes that form in response to exogenous microbial invasions and endogenous damage signals. Among the known inflammasomes, the activation of the NACHT (NAIP, CIITA, HET-E, and TP1 domain), leucine-rich repeat, and pyrin domain containing protein 3 (NLRP3) inflammasome is also primarily related to neuroinflammation and nerve cell damage. Previous studies reported that under the stimulation of dangerous signals like reactive oxygen species (ROS), the overexpression and interaction of thioredoxin-interacting protein (TXNIP) with NLRP3 may trigger the inflammatory response through the ROS/TXNIP/NLRP3 signaling pathway. This inflammatory response is the pathophysiological basis of some neurological and neurodegenerative diseases. The activation of inflammasome and apoptosis caused by TXNIP are widespread in brain diseases. Previous report has suggested the TXNIP/NLRP3 interaction interface. However, the comprehensive model of the TXNIP/NLRP3 interaction is still unclear. In this study, molecular docking experiments based on the existing crystal model of NLRP3 were performed to investigate the binding of TXNIP and NLRP3. Three *in silico* models of the TXNIP/NLRP3 complex were selected, and molecular dynamics simulations evaluated the binding stability of the possible interaction between the two proteins. The results revealed that the E690, E693, and D745 residues in NLRP3 and the K212 and R238 residues in TXNIP play a critical role in the TXNIP/NLRP3 interaction. N-terminal of TXNIP is essential in promoting the conformational changes of NLRP3, although it does not directly bind to NLRP3. Our findings reveal the possible binding mechanism between TXNIP and NLRP3 and the associated allosteric regulation of NLRP3. The constructed models may also be useful for inhibitor development targeting the TXNIP/NLRP3 interaction during inflammasome activation via the ROS/TXNIP/NLRP3 pathway.

## Introduction

Inflammasomes are complex cytoplasmic proteins that play an essential role in response to the invasion of pathogens and injury-related signals in immunity and inflammation (Kanneganti et al., 2007; Tschopp and Schroder, 2010; Lamkanfi and Dixit, 2014; Guo et al., 2015; Broz and Dixit, 2016). Among the known inflammasomes, NAIP, CIITA, HET-E, and TP1 domain (NACHT), leucine-rich repeat (LRR), and pyrin domain (PYD) domains-containing protein 3 (NLRP3) inflammasome has been extensively studied. The activation of NLRP3 inflammasome occurs in various nerve cells in the brain, including neurons, astrocytes, endothelial cells, and microglia (Li et al., 2015; Koka et al., 2017). As the core protein of the NLRP3 inflammasome, NLRP3 can act as the sensor component while its N-terminal PYD interacts with the apoptosis-associated speck-like protein containing a CARD (ASC) through the PYD–PYD interactions. The caspase recruitment domain of ASC recruits caspase-1 via interactions between the caspase recruitment domains to promote caspase oligomerization and activation, subsequently triggering the cleavage of the proinflammatory cytokines interleukin (IL)-1β and IL-18. NLRP3 simultaneously undergoes oligomerization, forming “specks” in the process (Stutz et al., 2017; Shim and Lee, 2018; Lopez-Castejon, 2020). However, the mechanism underlying the change of NLRP3 from the inactive to active state during oligomerization is unclear.

Various pathogen-associated molecular patterns and damage-associated molecular patterns can induce the NLRP3 inflammasome activation. Thioredoxin (TRX)-interacting protein (TXNIP) or thioredoxin-binding protein-2 (TBP-2), which is crucial in many signaling pathways, is a member of the α-arrestin family of proteins that acts as a regulator in various diseases, cell metabolism, transduction, and inflammation (Goldberg et al., 2003; Farrell et al., 2010; Schroder et al., 2010; Nishizawa et al., 2011; Tsubaki et al., 2020; Xie et al., 2020). Danger signals, such as reactive oxygen species (ROS), can reportedly promote TXNIP expression; and in turn, overexpression of TXNIP can stimulate the activation of the NLRP3 inflammasome (Schulze et al., 2006; Saxena et al., 2010; Zhou et al., 2010; Yoshihara et al., 2014; Yoshihara, 2020; Joaquim et al., 2021). Inflammasome activation via the ROS/TXNIP/NLRP3 signaling pathway is often associated with acute neurological and neurodegenerative diseases (Ishrat et al., 2015; Li et al., 2019; Ding et al., 2020; Gamdzyk et al., 2020). Previous studies reported that TXNIP can dissociate from the TXNIP–TRX complex and directly bind to NLRP3 under high ROS levels (Zhou et al., 2010). Therefore, exploring the direct interaction between TXNIP and NLRP3 is essential for studying neurological diseases related to the ROS/TXNIP/NLRP3 signaling pathway.

**FIGURE 1:**
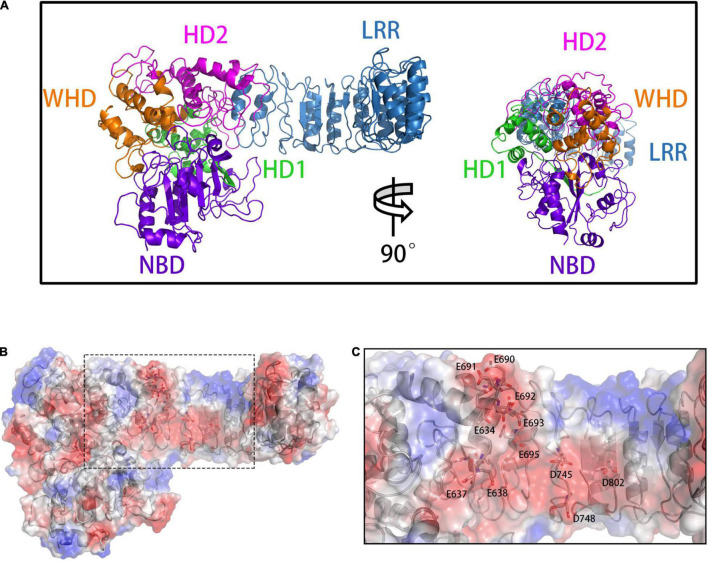
**(A)** The front (left) and side (right) views of the overall NLRP3 structure. The domains are presented in different colors: NBD (violet), HD1 (green), WHD (orange), HD2 (magenta), and LRR (blue). **(B)** Calculation of vacuum electrostatics on the surface of NLRP3. The area enclosed in a black rectangle indicate the negatively charged surface of NLRP3. **(C)** Key glutamate (E) and aspartate (D) residues involved in the formation of the negatively charged NLRP3 surface.

NLRP3 consists of an N-terminal PYD (residues 1–136), NACHT (residues 137–697), and an LRR region (residues 698–1034) (Sharif et al., 2019). NACHT can also be divided into the nucleotide-binding-domain (NBD, residues 137–359), helical domain 1 (HD1, residues 360–438), winged-helix domain (WHD, residues 439–534), and helical domain 2 (HD2, residues 535–697) (Figure 1A). On the other hand, TXNIP consists of the N-terminal (N-TXNIP, residues 8–147) and C-terminal (N-TXNIP, residues 154–299) arrestin domains. TXNIP reportedly binds to the LRR and NACHT of NLRP3, whereas C-TXNIP has a binding preference to NLRP3 (Zhou et al., 2010). However, the TXNIP–NLRP3 binding information and the effect of TXNIP binding on the NLRP3 structure are unclear. Hence, we hypothesized that C-TXNIP and NLRP3 directly bind together, which induces a change in the global conformation of NLRP3. To further verify this, we constructed *in silico* models of TXNIP docked with NLRP3 and evaluated the interaction between TXNIP and NLRP3 through molecular dynamics simulations. These models provide new structural insights into the TXNIP/NLRP3 interaction and the process underlying the change of NLRP3 from the inactive to active state during oligomerization. Our research may provide potential targets for inhibitor development based on the ROS/TXNIP/NLRP3 signaling pathway, which may be useful for future therapeutic studies on neuroinflammation and related neurological diseases.

## Results

### Regions of Opposite Charge on the Surfaces of NLRP3 and C-TXNIP Are the Basis for Binding

**FIGURE 2:**
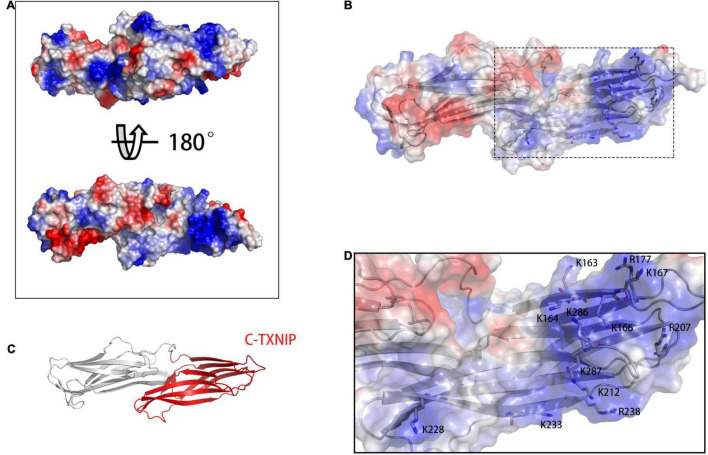
**(A)** Calculation of vacuum electrostatics on the front (top) and back (bottom) surfaces of TXNIP. **(B)** The area enclosed in a black rectangle indicate the positively charged surface of C-TXNIP. **(C)** Protein model of TXNIP, with C-TXNIP indicated in red. **(D)** Key lysine (K) and arginine (R) residues involved in the formation of the positively charged surface of TXNIP. Red-, white-, and blue-colored areas represent the negatively, neutrally, and positively charged surfaces of the protein, respectively.

**FIGURE 3:**
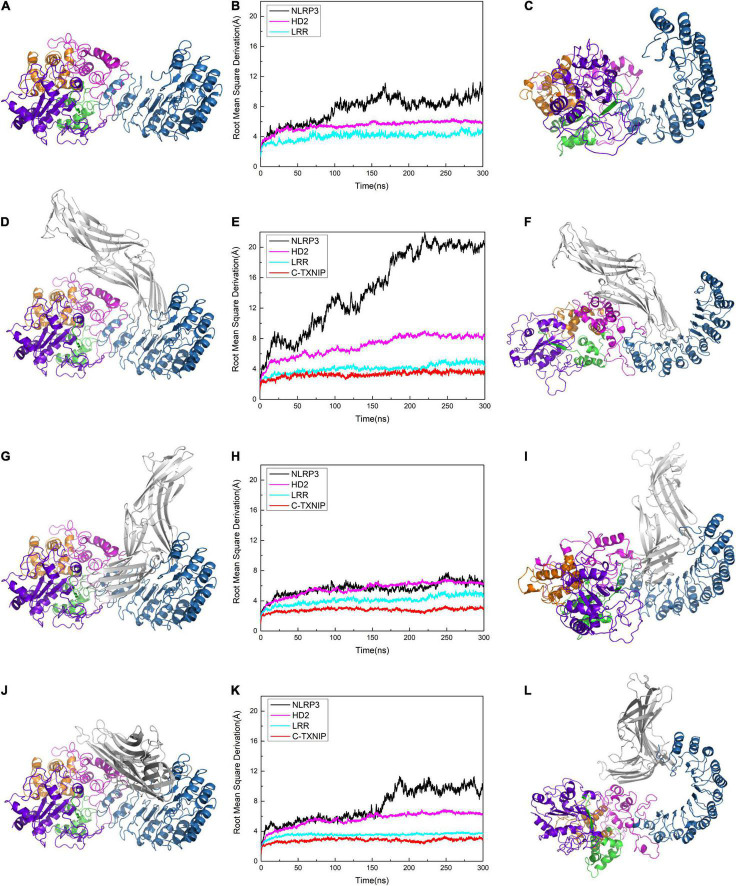
Protein conformation of **(A)** NLRP3 alone. Protein conformations of the NLRP3/TXNIP complexes in **(D)** Model 1, **(G)** Model 2, and **(J)** Model 3. Root mean square derivation (RMSD) curves of **(B)** NLRP3 alone, **(E)** Model 1, **(H)** Model 2, and **(K)** Model 3 after simulation at 0, 50, 100, 200, 250, and 300 ns. The black, magenta, cyan, and red curves represent the RMSD curves of the overall structure of NLRP3, NLRP3/HD2, NLRP3/LRR, and NLRP3/C-TXNIP, respectively. The last frame of the trajectory of **(C)** NLRP3 alone, **(F)** Model 1, **(I)** Model 2, and **(L)** Model 3 are shown.

To build the initial models, we calculated the vacuum electrostatics on the surfaces of NLRP3 and TXNIP. We discovered that an area was densely covered by negative charges in the HD2 and LRR of NLRP3 and contained glutamic acid (E) and aspartic acid (D) residues, including E634, E637, E638, D646, E675, E690, E691, E692, E693, E695, D700, D745, D748, D802, and D805 (Figures 1B,C). On the surface of C-TXNIP, a region containing lysine (K) and arginine (R) residues, namely K163, K164, K166, K167, R177, R207, K212, K228, K233, R238, K286, and K287, was densely covered with positive charges (Figures 2A–C). Notably, the surface areas of the two were large, and the charge density per area was high and concentrated. We speculated that the two high-density surfaces with opposite charges facilitate the stable binding between NLRP3 and TXNIP (Figure 2D). To confirm this, we used Z-DOCK program to construct theoretical NLRP3/TXNIP binding models. Among the top 10 of 100 outputs, we selected three with the most representative conformations and highest scores for subsequent calculations (Figures 3D,G,J). Based on the Z-DOCK scores, we designated complexes 1, 3, and 4 as Models 1, 2, and 3, respectively.

### C-TXNIP Triggers a Conformation Change in NLRP3 After Binding at a Specific Interface

To test the stability of the constructed models, we performed plain molecular dynamics simulations for Models 1–3 and a separate simulation for NLRP3 alone. Each model reached equilibrium after approximately 200 ns. In the model of NLRP3 alone, NLRP3 reached equilibrium after 100 ns (Figures 3A–C). In Model 1, the root mean square deviation (RMSD) of NLRP3 in the first 200 ns showed a jump of approximately 15 Å and reached equilibrium at approximately 200 ns (Figure 3E). This suggests that compared to unbound NLRP3, the conformation of NLRP3 greatly changed after binding to TXNIP and reached equilibrium after the simulation. By contrast, the RMSD of NLRP3 reached equilibrium after approximately 25 ns in Model 2 and remained stable after (Figure 3H), indicating that NLRP3 had a relatively stable conformation after binding to TXNIP. In Model 3, the RMSD of NLRP3 showed a slight jump (approximately 5 Å) between 150–200 ns and reached equilibrium after 200 ns (Figure 3K). This implies that NLRP3 underwent a moderate conformational change after binding to TXNIP. We selected the last frame of the trajectory of each model and recreated the models in a different interface to reflect the changes that occurred in NLRP3 after interacting with TXNIP (Figures 3F,I,L). Notably, compared to the initial conformation, Model 1 underwent a rotation of approximately 90° in the NBD, HD1, and WHD, Model 2 showed almost no change, and Model 3 had a slightly changed conformation. Overall, these results suggest that the TXNIP/NLRP3 binding in the Model 1 interface caused the considerable structural change of NLRP3, providing a potential basis for subsequent inflammasome assembly.

### Key Residues May Play Essential Roles in the TXNIP/NLRP3 Interaction

**FIGURE 4:**
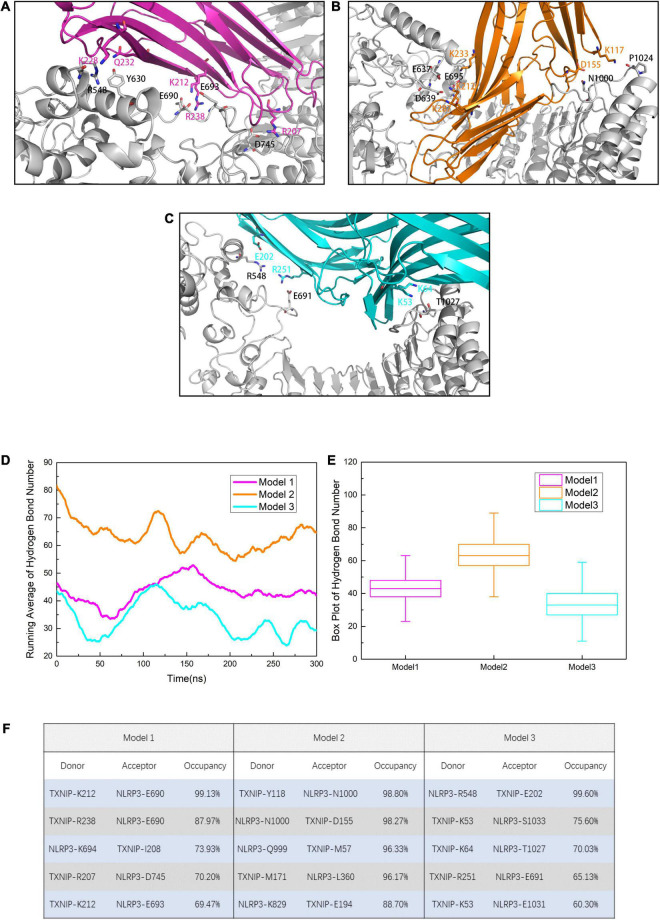
Residues involved in the intermolecular polar contacts of the NLRP3/TXNIP complexes in **(A)** Model 1, **(B)** Model 2, and **(C)** Model 3. The residues of NLRP3 are indicated in black, while the residues of TXNIP are indicated in magenta/orange/cyan. **(D)** Running average hydrogen bond number statistics of Models 1, 2, and 3 after the 300 ns simulation (each point is the average of nearby 100 frames). **(E)** The box plot of hydrogen bond number. Whiskers showed the outlier number with a coefficient of 1.5 and the boxes ranged from 25% to 75%. **(F)** The top five hydrogen bond occupancy, donors, and acceptors of Models 1, 2, and 3.

To identify the key residues in NLRP3/TXNIP binding and to explore the possible reason for the differences in the RMSD curves, we investigated the polar contacts and analyzed the hydrogen bond statistics in the models. Due to the different interface, the polar interactions of NLRP3/TXNIP in the three models were also relatively different (Figures 4A–C). In Models 1 and 2, the K212 of TXNIP was found to participate in the polar interaction with NLRP3. In Models 2 and 3, the R548 of TXNIP and LRR of NLRP3 participate in the polar interaction between TXNIP and NLRP3, respectively. In addition, several polar residues, namely the E690, E693, and D745 of NLRP3 and the K212 and R238 of TXNIP, may play essential roles in the TXNIP/NLRP3 interaction in Model 1. Notably, some neutral residues may also contribute to the polar contacts, such as the N1000/T1027 of NLRP3. We also analyzed the hydrogen bond statistics since hydrogen bonds are critical for intermolecular interactions. The running average hydrogen bond number and the top five residues that paired with high hydrogen bond occupancy were successfully determined (Figures 4D–F). The three models exhibited relatively stable hydrogen bonds, with the lowest hydrogen bond occupancy >30%. Furthermore, Model 2 possessed the highest total number of hydrogen bonds, whereas Model 3 had the lowest value. The most negligible hydrogen bond occupancy of Model 2 was approximately 90%, whereas that of Model 3 was approximately 60%. Taken together, these results suggest that not only hydrogen bonds but also key amino acid residues play an important function in the TXNIP/NLRP3 binding.

### The Flexibility of the HD2 of NLRP3 Increases After Binding to C-TXNIP

**FIGURE 5:**
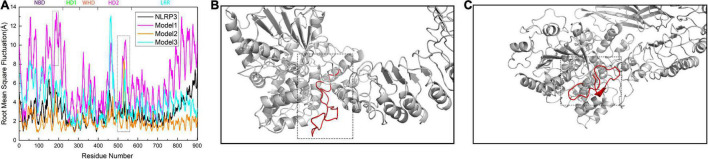
**(A)** Calculation of root mean square fluctuation (RMSF) for the residues of NLRP3 in the NLRP3/TXNIP interaction per model. The RMSF values of NLRP3 alone, Model 1, Model 2, and Model 3 are presented as black, magenta, orange, and cyan curves, respectively. The NLRP3 domains are indicated at the top. **(B)** The structure of approximately 30 residues of HD2 is marked in red. **(C)** The structure of amino acid residues 177–200 of NLRP3 is marked in red.

To further investigate the changes in the global structure of NLRP3 after binding to TXNIP, we calculated the root mean square fluctuation (RMSF) of NLRP3 in the models. We observed an overall increase in the RMSF of NLRP3 in Models 1 and 3 after binding to TXNIP (Figure 5A). By contrast, the RMSF in Model 2 decreased compared to Model 1. Notably, the RMSF value of NLRP3 in Model 1 significantly increased after binding to TXNIP, implying a significant change in the NLRP3 conformation. In addition, a region of approximately 30 residues were detected for the HD2 of NLRP3 (Figure 5B). The RMSF value of this region was low in unbound NLRP3. However, the RMSF value significantly increased after TXNIP was loaded in the three models. In addition, we observed that the RMSF values of residues 177–200 in NLRP3 increased in Model 1 (Figure 5C), whereas no similar changes were observed in Models 2 and 3.

### Correlation Factor Analysis Suggests a Potential Correlation Between TXNIP and NLRP3

**FIGURE 6:**
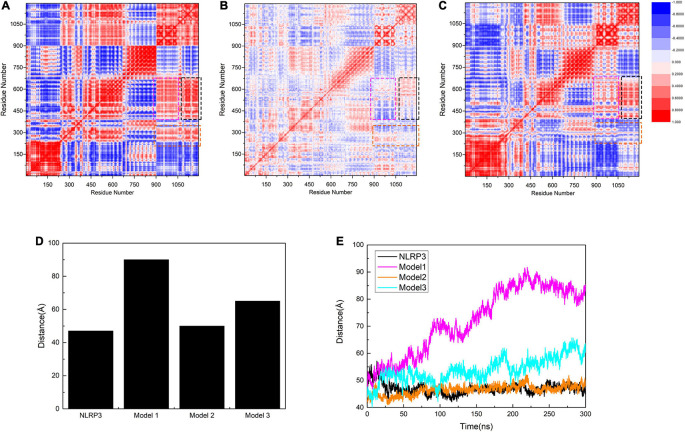
Correlation factor analysis results for 1,050 residues of **(A)** Model 1, **(B)** Model 2, and **(C)** Model 3. The black rectangle shows HD2 (residues 535–697 of NLRP3) and part of LRR (residues 698–832 of NLRP3) in relation to C-TXNIP (residues 154–299 of TXNIP). The magenta rectangle indicates HD2 and part of LRR corresponding to N-TXNIP (residues 8–147 of TXNIP). The orange rectangle presents HD1 (residues 360–438 of NLRP3) and part of WHD (residues 439–493 of NLRP3) corresponding to TXNIP. Residues with strong and weak correlations are represented in red and blue, respectively. The legend for the degree of correlation is presented on the right side. **(D)** The maximal distance (Å) between residues L338 and G779 in NLRP3 alone, Model 1, Model 2, and Model 3. **(E)** Distance curves (Å) between residues L338 and G779 in NLRP3 alone (black), Model 1 (magenta), Model 2 (orange), and Model 3 (cyan) after simulation at 0, 50, 100, 200, 250, and 300 ns.

We subsequently calculated the residue-to-residue correlation factors that reflect the vibration styles per residue in the three models. We identified the first area that showed positive values in Models 1 and 3, but has almost neutral values in Model 2 (enclosed in a black rectangle, Figures 6A–C). In Models 1 and 3, C-TXNIP demonstrated a strong correlation with the HD2 and partial correlation with the LRR of NLRP3, whereas, no similar correlations were detected in Model 2. Similarly, the second area (enclosed in a magenta rectangle) showed positive values in Models 1 and 3 and almost neutral values in Model 2. Furthermore, the correlation was weaker in Model 3 than in Model 1, suggesting that N-TXNIP has a relatively strong correlation with the HD2 and partial correlation with the LRR of NLRP3 in Model 1 and a weak correlation with both domains in Model 3. In particular, N-TXNIP and NLRP3 showed no direct correlation in Model 1. The third area (enclosed in an orange rectangle) had positive, slightly negative, and neutral values in Models 1, 3, and 2, respectively. These results suggest that TXNIP strongly correlated with the HD2 and partially correlated with the WHD of NLRP3 in Model 1, but only slightly correlated with both domains in Model 3. By contrast, these correlations were only slightly observed in Model 2. We also calculated the distance between the Cα atoms of L338 and G779 in NLRP3 to accurately describe the structural changes following the interaction with TXNIP. The distance in unbound NLRP3 was approximately 45 Å, which was almost similar to the distance in Model 2 (Figures 6D,E). In Model 3, the distance fluctuated to approximately 55 Å. Notably, in Model 1, the distance stabilized at approximately 85 Å after 200 ns, suggesting that the NLRP3 in Model 1 exhibited the most significant structural change.

### Free Energy Calculation Provides a Deeper Insight Into the NLRP3/TXNIP Interaction

**FIGURE 7:**
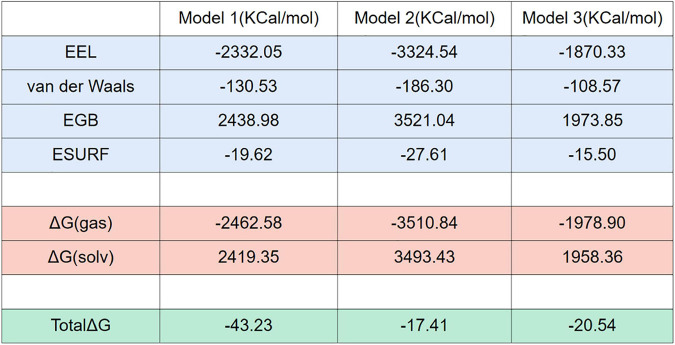
Free energy calculations and decompositions for each model. All units are in KCal/mol.

To further investigate the binding adaptability between NLRP3 and TXNIP and to determine the implicit relationship between molecules, we predicted the binding free energy of the three models in an inclusive solution. We intercept the last 100 ns (1,000 frames) that are relatively stable in 300 ns simulation for free energy calculation. NLRP3 was designated as the acceptor in the calculations, while TXNIP was considered as the donor. The results (all in KCal/mol) showed that Model 1 had the most significant decrease in binding energy, whereas the decrease in Models 2 and 3 was negligible (Figure 7), indicating that TXNIP is more energetically favorable to NLRP3. In all models, both electrostatic interaction energy (E_*EL*_) and van der Waals (vdW) contributed to the binding of TXNIP and NLRP3. However, E_*EL*_ had a more significant contribution than vdW, suggesting that the TXNIP/NLRP3 interaction is mainly dependent on polar contact. In particular, Model 2 contained a large E_*EL*_ and vdW, which suggests that its polar contact was the strongest. By contrast, vdW had a more significant contribution than E_*EL*_ in Models 1 and 3. The huge interaction area between TXNIP and NLRP3 in Model 2 may have contributed to the large E_*EL*_ and vdW observed.

### An Additional Model Reveals the Allosteric Effect of N-TXNIP on NLRP3

**FIGURE 8:**
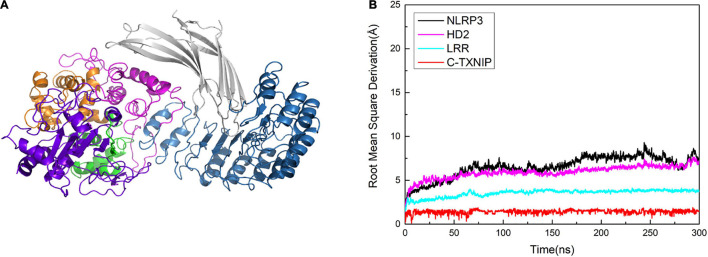
**(A)** Model of NLRP3 bound to TXNIP containing only C-TXNIP. **(B)** Root mean square derivation (RMSD) curves of NLRP3 (black), HD2 (magenta), LRR (cyan), and C-TXNIP (red) in this model after simulation at 0, 50, 100, 200, 250, and 300 ns.

To determine the role of N-TXNIP on the structural changes of NLRP3, we constructed an additional model and performed correlation factor analysis. In Model 1, we observed that although there was no direct interaction between N-TXNIP and NLRP3, the correlation between the two was strong. Therefore, we removed N-TXNIP and only retained C-TXNIP for subsequent molecular dynamics simulations. We discovered that C-TXNIP could not trigger the structural change of NLRP3 (Figures 8A,B). Furthermore, the overall RMSD curve of NLRP3 that interacted with only C-TXNIP was slightly lower compared to that of unbound NLRP3. This result implies that although N-TXNIP does not directly interact with NLRP3, it plays a vital role in the activation of the NLRP3 inflammasome.

## Discussion

In this work, we generated three theoretical NLRP3/TXNIP models and characterized them using vacuum electrostatic calculation, RMSD calculation, polar contact analysis, hydrogen bond analysis, RMSF calculation, correlation factor analysis, and free energy analysis to evaluate their structural characteristics. We also compared the data of TXNIP-free and TXNIP-bound NLRP3 in the three models to determine the effect of TXNIP on the structure of NLRP3 during inflammasome activation.

Based on our findings, the TXNIP of Model 1 has the best binding affinity in the NLRP3/TXNIP interaction among the three models. We consider Model 1 to be the best of the three models, and most likely to be the real TXNIP/NLRP3 binding. In addition, the RMSD and RMSF of NLRP3 in Model 1 indicated that after the binding, the oscillation degree of the NLRP3 global structure increased and its conformation underwent tremendous changes. We observed that this change was mainly due to the altered condition of NACHT from the “L” to the “I” type; additionally, NBD, HD1, and WHD underwent a rotation of approximately 90° (see Supplementary Material). This observation is consistent with the previously proposed model of the NEK7/NLRP3 crystal structure (Sharif et al., 2019). However, the existing hypothetical activation model was only based on the homologous structure of NLRP3 (NLRC4). In general, the conformational change reduces the steric hindrance on the side of NLRP3, which is conducive to further oligomerization (Hu et al., 2015; Zhang et al., 2015; Tenthorey et al., 2017; Yang et al., 2018). Notably, our molecular dynamics simulation on the TXNIP/NLRP3 model demonstrated similar features to the previous NEK7/NLRP3 model. Therefore, we hypothesize that the “L”-to-“I” type transition of NACHT is the structural basis of NLRP3 activation. In addition, D745 is involved in the binding of the two proteins in the previously reported NEK7/NLRP3 model. Similarly, D745 was also found to be a critical amino acid residue in our constructed TXNIP/NLRP3 model. This implies that D745 may play an essential function in the binding of NLRP3 to other molecules.

A previous study reported that intramolecular interactions between NACHT and LRR may be necessary for autoinhibition in the absence of activating signals (Manji et al., 2002). Our RMSF calculations revealed that after the conformational change of NLRP3, the flexibility of residues 177–200 on NLRP3 was enhanced. In the absence of conformational change, the flexibility was significantly greater in this segment. We speculate that this region may be involved in the autoinhibition of NLRP3 in the inactive state. In addition, although NLRP3 has some natural variants, the key polar residues we identified in the calculation, specifically E690, E693, and D745, did not change and were relatively conserved. This suggests that these amino acid residues on NLRP3 may be involved in the actual binding of TXNIP and NLRP3. Among the variants of TXNIP, a natural variant from R177 to Q177 was detected. In our calculations, R177 was a key polar residue in the binding interface of TXNIP. However, this amino acid residue is variable, and further study may lead to some significant findings on TXNIP/NLRP3 binding.

**FIGURE 9:**
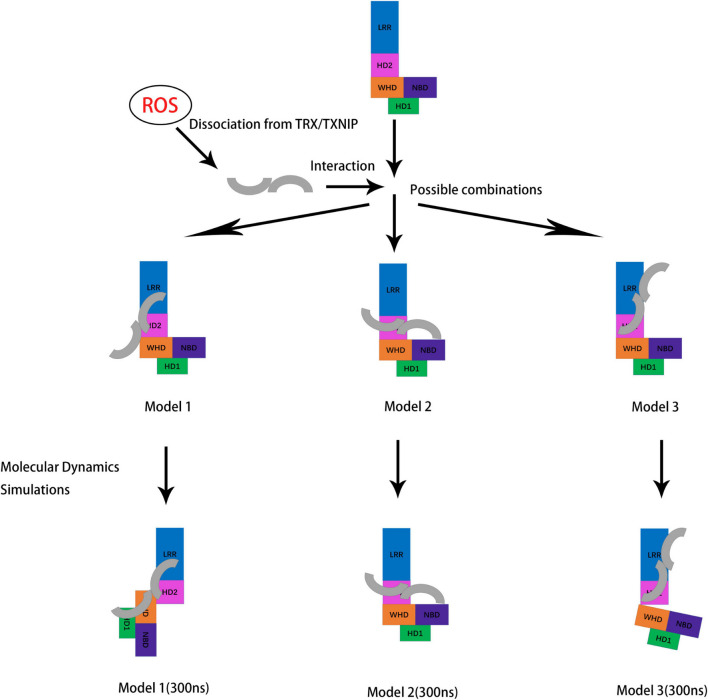
Schematic diagram showing the potential conformations of the three constructed models for NLRP3/TXNIP binding after molecular dynamics simulations. TXNIP dissociated from TRX under ROS stimulation. Then, TXNIP and NLRP3 were bound at the potential interface. Molecular dynamics simulations of the models were performed, and the outputs of the last frame of model trajectory were the final results.

Our work mainly focused on the interaction of TXNIP and NLRP3 and the overall changes in the structure of NLRP3 after the binding. Hence, the assembly of the NLRP3 inflammasome and the specific mechanism underlying NLRP3 oligomerization are still unclear. Interestingly, the PYD binding mechanism in the NLRP3/ASC has been previously predicted (Riaz et al., 2021). However, the complete activation mechanism of the PYD in NLRP3 requires further investigation. Since the activation of the NLRP3 inflammasome is critical in neuroinflammation and other inflammation-related events, virtual screening studies for NLRP3 inhibitors have emerged (Sebastian-Valverde et al., 2021). Research focused on the ROS/TXNIP/NLRP3 signaling pathway has also increased in recent years (Bai et al., 2021; Wang et al., 2021; Zhang et al., 2021). For example, knockdown experiments to block the upstream signaling of the TXNIP/NLRP3 signaling pathway or to inhibit the expression of TXNIP at the transcriptional level and consequently prevent the activation of the NLRP3 inflammasome may help treat various diseases involving inflammation. Notably, we identified several amino acid sites that may be potential inhibitor targets, such as K212 and K238. By blocking these key sites, we can either inhibit the binding of TXNIP and NLRP3 or lower the affinity of TXNIP for NLRP3, further interfering with the assembly of the downstream NLRP3 inflammasome to prevent inflammation. We also discovered that although N-TXNIP does not directly bind to NLRP3, our findings provide new possible directions and ideas for synthesizing other NLRP3/TXNIP binding inhibitors. However, our theoretical model has some limitations. Since there is no detailed crystal model currently available for TXNIP/NLRP3 binding, the specific allosteric mechanism regulating NLRP3 is still unclear. In conclusion, our work provides valuable information on the TXNIP/NLRP3 binding characteristics, activation and allostery of the NLRP3 inflammasome, and potential targets for inhibitor research that may be useful for future studies on neuroinflammation and various diseases associated with NLRP3 (Figure 9).

## Materials and Methods

### Molecular Docking and Model Building

The NLRP3 model was based on the cryogenic electron microscopy (cryo-EM) structure of NLRP3 that was bound to NEK7 (PDB code: 6NPY). We removed NEK7 and used Coot (Emsley and Cowtan, 2004) to complement the missing NACHT and LRR structures, with reference to the NLRP3 sequence. The TXNIP model was based on the structure of the TRX–TXNIP complex (PDB code: 4LL1). We removed TRX and completed the TXNIP structure based on the TXNIP sequence using Coot program. Z-DOCK program (version 3.0.2) was used for all docking studies (Pierce et al., 2011). Flexible residues were assigned to amino acids involved in the active sites of TXNIP/NLRP3. The potential energy of the system was minimized, and docking conformations were prepared using the genetic algorithm. For cluster analysis, a total of 100 potential conformations were exported. We extracted and aggregated the conformations with the highest Z-DOCK scores. The representative conformations were chosen after molecular dynamics simulations.

### Molecular Dynamics Simulations and Free Energy Calculations

All molecular dynamics simulations were performed using AMBER 20 program (Case et al., 2021), following the amber19sb all-atom force field parameters (Tian et al., 2020). Since the NLRP3/TXNIP complex is neutral, a total number of 52 Na^+^ and Cl^–^ ions were added to maintain a 100 mmol/L ionic strength, and each system was specifically solvated using the TIP3P water potential in the water box, with a minimum solute-wall-distance of 12 Å. The following procedures were performed for all simulations. First, the potential energy of the entire system was lowered to eliminate unfavorable connections. Four rounds of minimization, with a total of 2,500 steps, were conducted. In the first two rounds, which employed the steepest descent (SD) and conjugate gradient (CG) minimization methods, respectively, the entire system was restricted (except for the water and ions). Then, the system was uncontrolled in the last two rounds. For non-bounded interaction, the cut-off distance was set at 12 Å, and the SHAKE algorithm was used to constrain the bonds containing hydrogen atoms. Second, the energy-minimized structure was heated to over 200 ps from 0 to 300 K (with a temperature coupling of 0.2 ps) in 1 bar, while the atom positions of the protein were restrained to a small value of 10 kcal/(mol×Å^2^). A 2-fs integration step was used. Finally, conventional molecular dynamics simulation was performed for 300 ns without the restrains. Simulation of apo-NLRP3 model was performed by the similar approaches to observe the conformational changes of unloaded NLRP3. Only 42 Cl^–^ were added to keep the solution neutral and other steps followed a similar pattern as above.

Free energy calculations were conducted using the MMPBSA.py script in AmberTools (version 21), following the GBSA implicit solvent model. We intercepted the last 1,000 frames (100 ns) of the total 3,000 frames (300 ns) for free energy calculation. Free energy calculation interval were set as 2 and a total of 500 frames were used to obtain the final free energy calculation result. The ionic strength of the system was fixed to 100 mmol/L. The reported results are in KCal/mol. Other parameters were set in accordance with previously published methods (Wang and Hou, 2020).

### Fluctuation and Correlation Analyses

The RMSF values of the residues were used to quantify the fluctuations and flexibilities of the Cα atoms in the protein backbone along the trajectory decomposed into residues, in comparison to the average structures. The RMSF_*i*_ value of the Cα atom per residue was determined as follows:


$$R\,M\,S\,F_{i}=\sqrt{\frac{\sum_{t=1}^{T}{\left(r_{i}\,\left(t\right)-\left(r_{i}\right)\right)}^{2}}{T}}$$

where *T* denotes the number of snapshots in the trajectory, *r_*i*_(t)* denotes the position of the Cα atom in residue *I* at time *t*, and *r*_*i*_ denotes the time-averaged position of the Cα atom.

The dynamic properties of the protein and the amount to which motions in distinct regions of the protein are correlated were determined using the cross-correlation coefficients or C(i,j):


$$C\,\left(i,j\right)=\frac{\left(\Delta \,r_{i}\times \Delta \,r_{j}\right)}{\sqrt{\left(\Delta \,r_{i}^{2}\,\Delta \,r_{j}^{2}\right)}}$$

where Δr*i* and Δr*j* represent the displacement vectors of the Cα atoms in residues *i* and *j*, respectively and the angle brackets represent the ensemble averages. The correlation coefficients of the protein sections were averaged, and the resulting C(i,j) values were presented as a two-dimensional graph. All structural analyses were performed using the CPPTRAJ script (Roe and Cheatham, 2013).

## Data Availability Statement

The original contributions presented in the study are included in the article/Supplementary Material, further inquiries can be directed to the corresponding author.

## Author Contributions

FC and NW: conceptualization and validation. NW: methodology, software, investigation, writing—review and editing, project administration, funding acquisition, and resources and data curation. FC: formal analysis, writing—original draft preparation, and visualization. All authors have read and agreed to the published version of the manuscript.

## Conflict of Interest

The authors declare that the research was conducted in the absence of any commercial or financial relationships that could be construed as a potential conflict of interest.

## Publisher’s Note

All claims expressed in this article are solely those of the authors and do not necessarily represent those of their affiliated organizations, or those of the publisher, the editors and the reviewers. Any product that may be evaluated in this article, or claim that may be made by its manufacturer, is not guaranteed or endorsed by the publisher.
