# Supplementary figures and images for: N-Lobe of TXNIP Is Critical in the Allosteric Regulation of NLRP3 via TXNIP Binding
Source: Front Aging Neurosci. 2022 Jun 2;14:893919. doi: 10.3389/fnagi.2022.893919 (PMC9201253; doi:10.3389/fnagi.2022.893919)

Docking results that have a similar conformation with model 1, 2, 3 by PYDOCK:


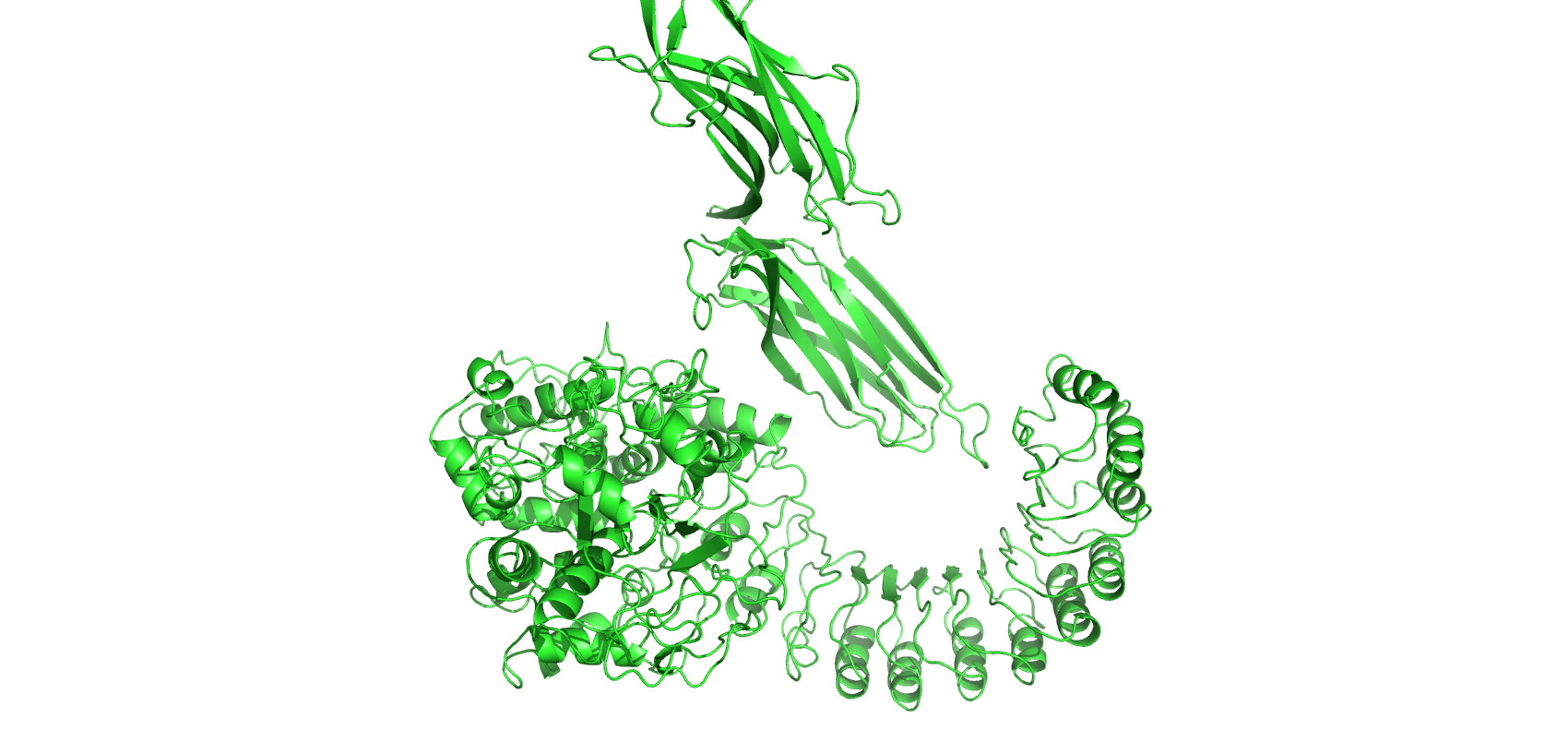

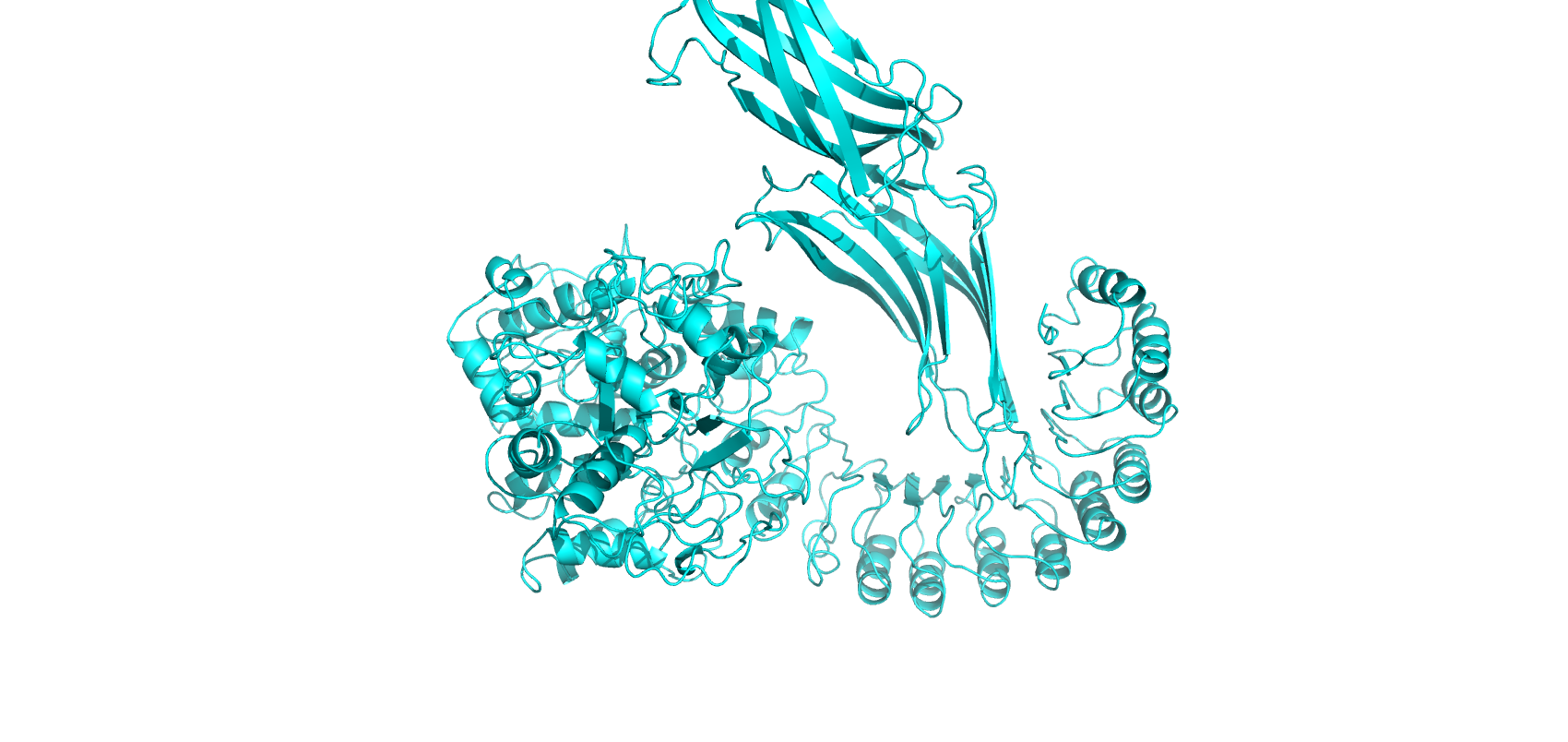

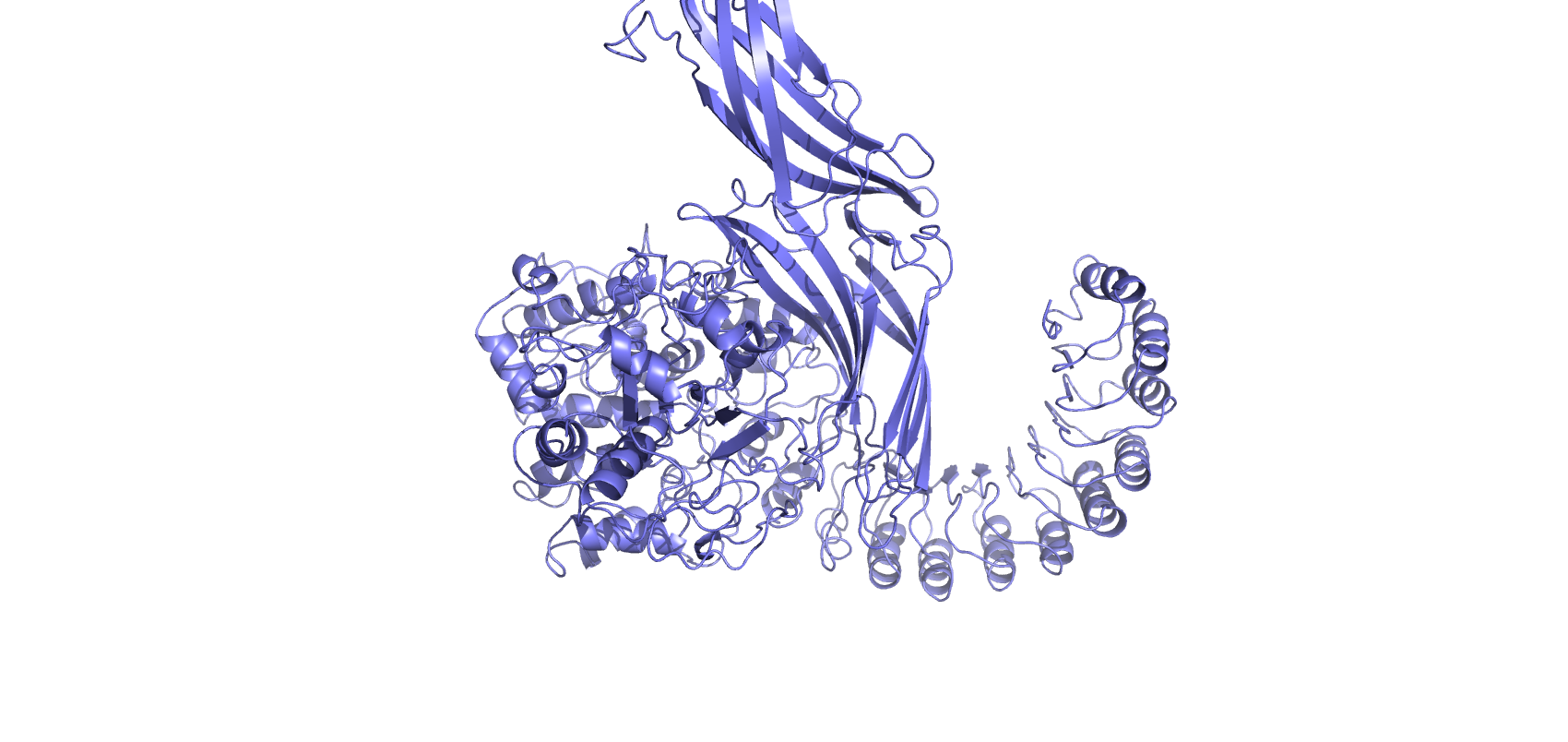

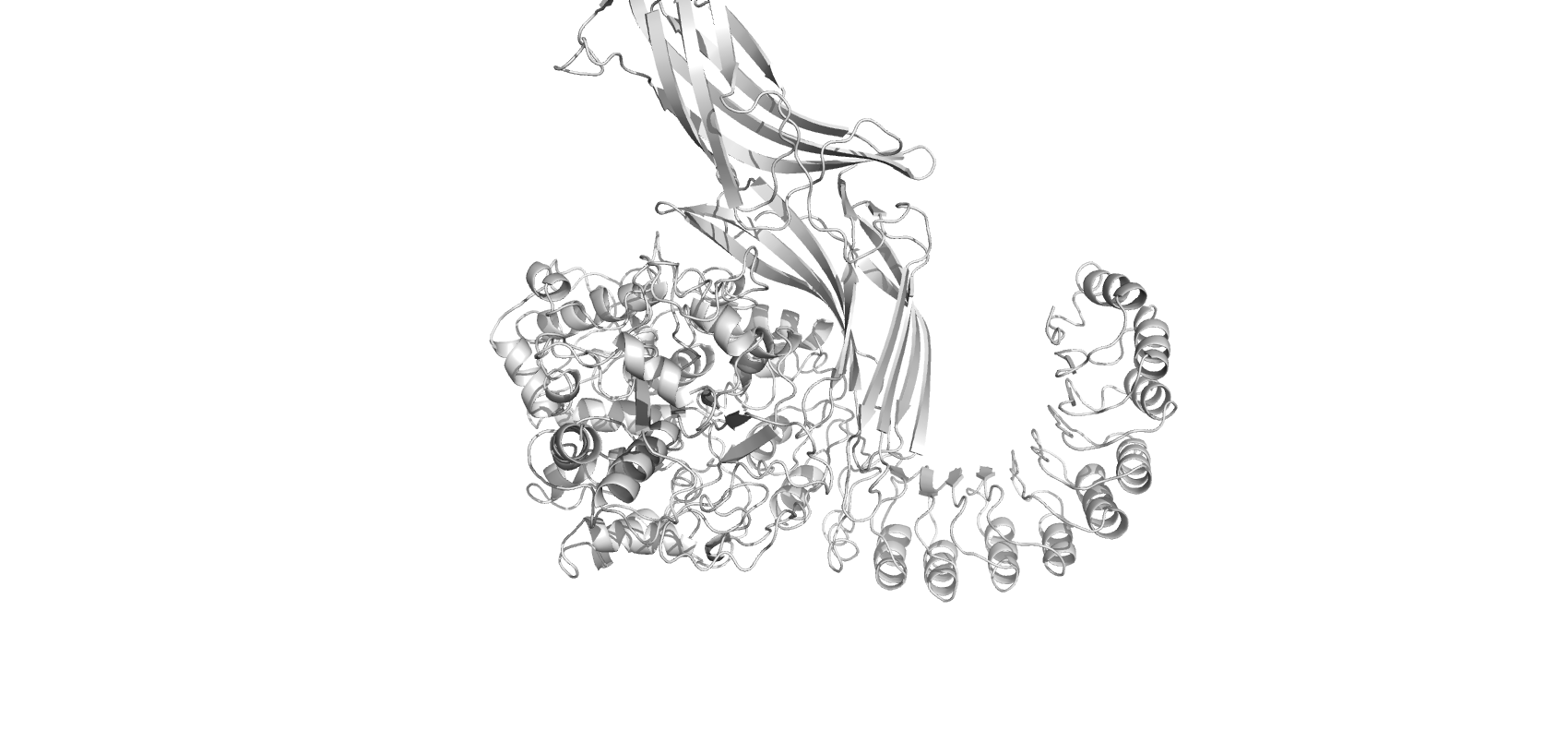

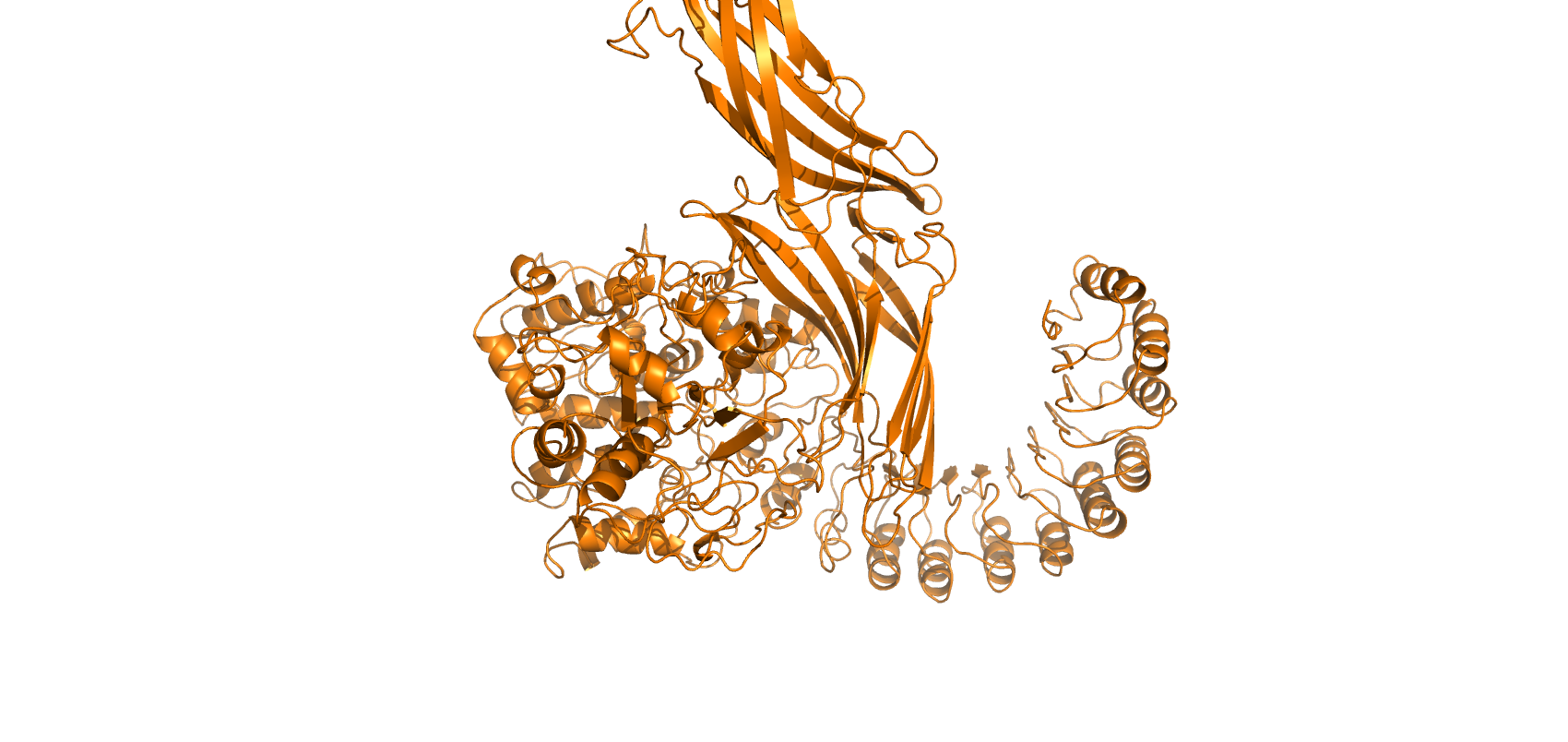

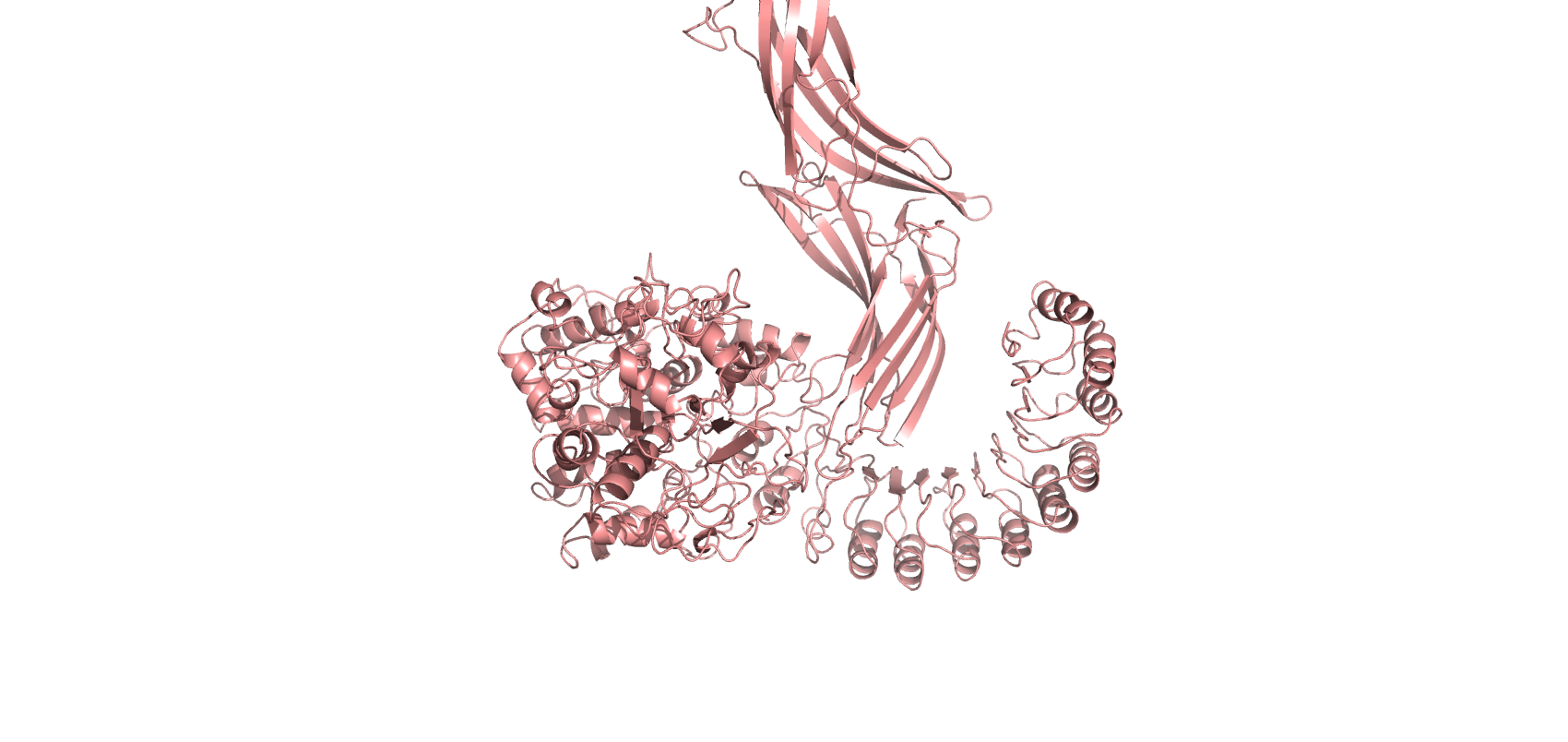

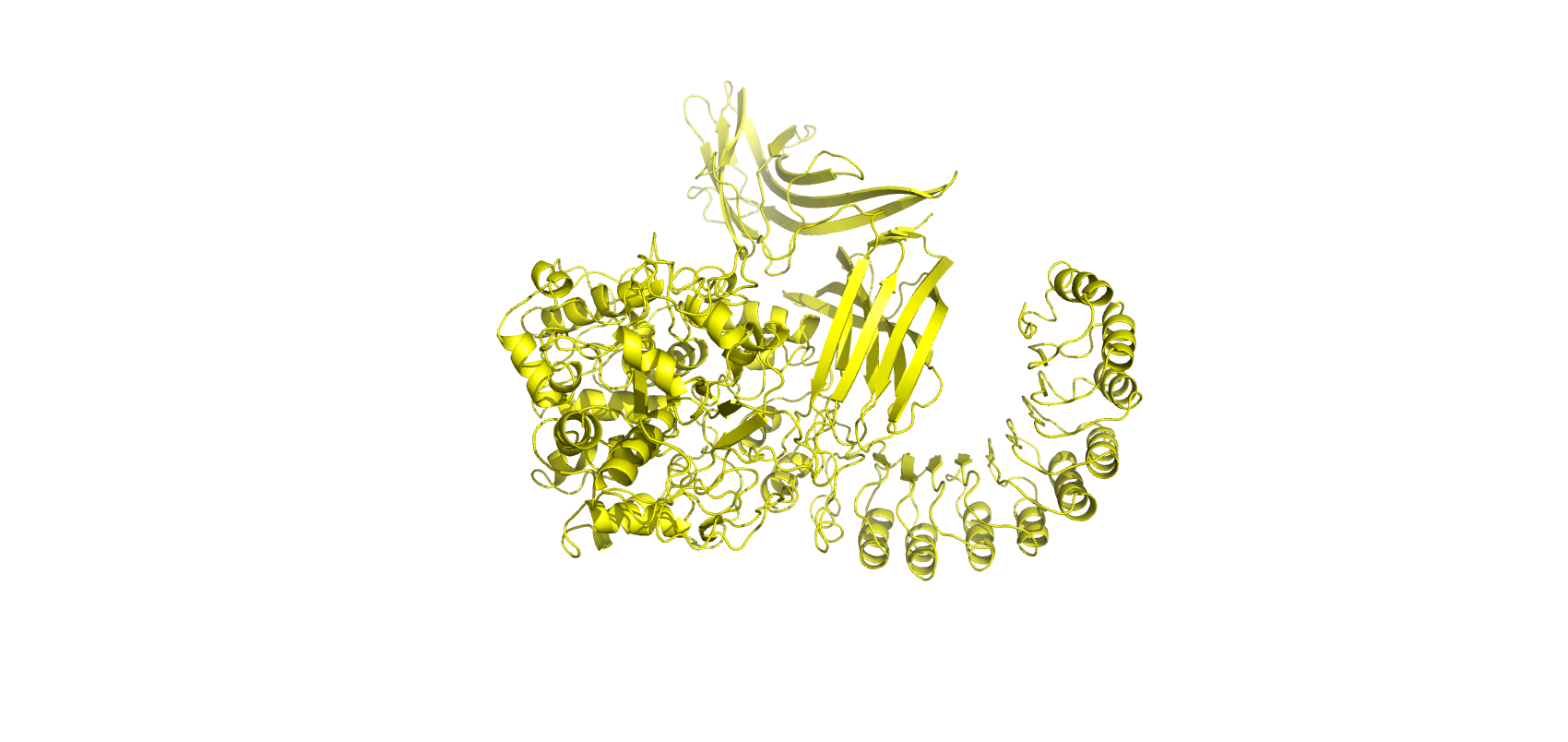

Supplement: Supplementary file 1 [file Table_1.DOCX]
